# Mechanistic study of pre-eclampsia and macrophage-associated molecular networks: bioinformatics insights from multiple datasets

**DOI:** 10.3389/fgene.2024.1376971

**Published:** 2024-05-23

**Authors:** Jinfeng Cao, Wenxin Jiang, Zhe Yin, Na Li, Chao Tong, Hongbo Qi

**Affiliations:** ^1^ Department of Obstetrics, The First Affiliated Hospital of Chongqing Medical University, Chongqing, China; ^2^ Chongqing Key Laboratory of Maternal and Fetal Medicine, Chongqing Medical University, Chongqing, China; ^3^ Joint International Research Laboratory of Reproduction and Development of Chinese Ministry of Education, Chongqing Medical University, Chongqing, China; ^4^ Department of Obstetrics and Gynecology, Women and Children’s Hospital of Chongqing Medical University, Chongqing, China

**Keywords:** pre-eclampsia, macrophages, immune infiltration, GSEA, CMAP

## Abstract

**Background:**

Pre-eclampsia is a pregnancy-related disorder characterized by hypertension and proteinuria, severely affecting the health and quality of life of patients. However, the molecular mechanism of macrophages in pre-eclampsia is not well understood.

**Methods:**

In this study, the key biomarkers during the development of pre-eclampsia were identified using bioinformatics analysis. The GSE75010 and GSE74341 datasets from the GEO database were obtained and merged for differential analysis. A weighted gene co-expression network analysis (WGCNA) was constructed based on macrophage content, and machine learning methods were employed to identify key genes. Immunoinfiltration analysis completed by the CIBERSORT method, R package “ClusterProfiler” to explore functional enrichment of these intersection genes, and potential drug predictions were conducted using the CMap database. Lastly, independent analysis of protein levels, localization, and quantitative analysis was performed on placental tissues collected from both preeclampsia patients and healthy control groups.

**Results:**

We identified 70 differentially expressed NETs genes and found 367 macrophage-related genes through WGCNA analysis. Machine learning identified three key genes: FNBP1L, NMUR1, and PP14571. These three key genes were significantly associated with immune cell content and enriched in multiple signaling pathways. Specifically, these genes were upregulated in PE patients. These findings establish the expression patterns of three key genes associated with M2 macrophage infiltration, providing potential targets for understanding the pathogenesis and treatment of PE. Additionally, CMap results suggested four potential drugs, including Ttnpb, Doxorubicin, Tyrphostin AG 825, and Tanespimycin, which may have the potential to reverse pre-eclampsia.

**Conclusion:**

Studying the expression levels of three key genes in pre-eclampsia provides valuable insights into the prevention and treatment of this condition. We propose that these genes play a crucial role in regulating the maternal-fetal immune microenvironment in PE patients, and the pathways associated with these genes offer potential avenues for exploring the molecular mechanisms underlying preeclampsia and identifying therapeutic targets. Additionally, by utilizing the Connectivity Map database, we identified drug targets like Ttnpb, Doxorubicin, Tyrphostin AG 825, and Tanespimycin as potential clinical treatments for preeclampsia.

## 1 Background

Pre-eclampsia (PE) is a common hypertensive disorder occurring typically after 20 weeks of pregnancy, characterized primarily by elevated blood pressure (>140/90 mmHg, 1 mmHg = 0.133 kPa) and proteinuria (>300 mg/L). It is a major cause of morbidity and mortality for both the perinatal period and pregnant women and newborns worldwide, affecting 2%–8% of pregnancies, particularly in low- and middle-income countries (Yang et al., 2013; González-Garrido et al., 2017). Epidemiological studies have shown that 5%–7% of pregnant women worldwide suffer from this disease, posing a significant threat to the life and health of pregnant women and newborns (Kuc et al., 2011; Firoz et al., 2022). Therefore, understanding the risk factors of PE is crucial for its prevention and treatment.

As is well known, PE originates from placental dysfunction, characterized by fundamental pathophysiological changes such as endothelial injury, systemic arterial spasm, inadequate invasion of maternal spiral arteries by placental trophoblast cells, and alterations in placental development, perfusion, and nutrient transport (Founds et al., 2018). However, the exact etiology and pathogenesis of PE remain incompletely understood, making it a critical research topic in obstetrics. Many studies have shown that the pathogenesis of PE may be related to the interaction of various genetic and environmental factors (ACOG, 2019; ACOG, 2020; Cunningham, 2021)For example, the placenta relies heavily on energy provided by mitochondria, and mitochondrial damage caused by circulating bioactive factors released from the placenta may lead to endothelial dysfunction and subsequent maternal hypertension (Xu et al., 2019; Hu and Zhang, 2022). Additionally, PE may occur in pregnant women with underlying conditions such as hypertension or other high-risk factors like multiple pregnancies and advanced maternal age (Gao et al., 2020; Saito et al., 2023).

Systemic vascular dysfunction in the mother forms the basis of the clinical features of PE. Severe complications such as HELLP syndrome and placental abruption, if left untreated, can be life-threatening for PE patients (Marshall et al., 2018). The only known treatment for PE is the delivery of the fetus and placenta (Brown, 2020). Maternal-fetal immune tolerance is a complex phenomenon, with semi allogeneic fetuses exposed to maternal immune tolerance for up to 10 months during pregnancy. This crucial immune phenomenon involves three maternal-fetal interfaces (Bürk et al., 2001): firstly, maternal uterine decidual cells interact with fetal trophoblasts; secondly, the decidua is located in the maternal part of the placenta and is infiltrated by fetal extravillous trophoblast cells; thirdly, the villous chorion is the main component of the placental cells, formed by fetal-derived syncytiotrophoblast cells that directly interact with maternal blood circulation. Therefore, a healthy mother needs to develop immune tolerance to avoid immune attacks on fetal tissues while retaining the ability to defend against pathogens. At these three interfaces, several mechanisms of immune cell regulation have been elucidated (Escribese et al., 2012), and it is known that decidual macrophages in the maternal decidua possess innate immune function (Yang et al., 2017). Macrophages are well-known antigen-presenting cells that may play a role in immune regulation through phagocytosis and cytokine secretion. During pregnancy, decidual macrophages are associated with the secretion of immune-suppressive cytokines, maintaining immune tolerance (Fujihara et al., 2003). Macrophages, such as Th1 and Th2 lymphocytes, can be classified into different types based on their distinct functions. M1 macrophages are involved in the classical functions of host defense against foreign pathogens, while M2 macrophages participate in immune regulation and tolerance (Schonkeren et al., 2011). In the decidua, maternal macrophages expressing DC-SIGN exhibit a CD14+/CD163+ phenotype and are considered M2 macrophages (Kim et al., 2007).

Increasing evidence suggests that various genes and cellular pathways are involved in the occurrence and development of PE (Chelbi and Vaiman, 2008). Therefore, high-throughput platforms such as microarrays are increasingly used for the analysis of miRNA and gene expression in PE (Luo et al., 2017). Many recent studies have explored the pathogenesis of PE using various bioinformatics tools. The VEGF signaling pathway is believed to be most related to PE. The VEGF, FLT1 and elements involved in arginine metabolism, including NO production, are considered the main mechanisms by which the placenta participates in PE (Noris et al., 2005; Cindrova-Davies et al., 2011; Sundrani et al., 2013). Additionally, microRNAs are associated with PE by targeting key genes that regulate the differentiation of human trophoblast cells (Zhu et al., 2015; Zhang et al., 2016). Given the importance of immune cell infiltration in the pathogenesis of PE, analysis of immune cell infiltration can be used to select hub genes in PE as molecular markers for different subtypes of PE, providing new insights into the mechanism of PE and potential biomarkers for its diagnosis and treatment. In this study, we discussed the relationship between immune cell infiltration and PE based on WGCNA screening and GSEA analysis, as well as the differences in immune cell infiltration between PE and healthy pregnant women. We identified hub genes for PE molecular subtyping. The differences in hub genes between different PE subtypes suggest that these genes may serve as potential markers for PE subtyping, providing new insights into the pathogenesis of PE and potential biomarkers for its diagnosis and treatment.

In this study, we discussed the relationship between immune cell infiltration and pre-eclampsia based on WGCNA screening and GSEA analysis. Through these methods, we were able to gain valuable insights into the distinct patterns of immune cell infiltration observed in women with pre-eclampsia compared to their healthy counterparts during pregnancy. We identified hub genes for PE molecular subtyping. These genes exhibit significant differences across various PE subtypes, indicating their potential as markers for PE subtyping. This novel finding not only sheds new light on the pathogenesis of PE but also opens up possibilities for the development of more precise diagnostic and prognostic biomarkers, paving the way for improved clinical management and treatment strategies for this complex disorder.

## 2 Materials

### 2.1 Clinical sample collection

The samples used in this study were all obtained from the First Affiliated Hospital of Chongqing Medical University. Placental tissues from seven pregnant women diagnosed with PE and seven normal pregnant women were collected immediately after cesarean section. Individuals with diabetes, kidney disease, metabolic syndrome, infections, chromosomal abnormalities, or structural anomalies were excluded from the study. All patients underwent elective cesarean section, and they were informed and signed informed consent forms. Approximately 1 cm^2^ of placental tissue was collected immediately after delivery, washed, and divided. A portion was snap-frozen in liquid nitrogen, another portion was placed in RNA storage solution, and both were stored long-term at −80°C. The remaining portion was fixed in 4% paraformaldehyde and embedded in OCT. This study followed the principles outlined in the Helsinki Declaration and obtained approval from the Research Ethics Committee of the First Affiliated Hospital of Chongqing Medical University.

### 2.2 Data acquisition

Details are provided in Supplementary Table S1. The Series Matrix File data file of GSE75010 were downloaded from the NCBI GEO public database. The annotation file is GPL6244. A total of expression profile data of 157 patients were included, including 77 in the control group and 80 in the disease group. The Series Matrix File data file of GSE74341 were downloaded from the NCBI GEO public database. The annotation file is GPL16699. A total of expression profile data of 25 patients were included, including 10 cases in the control group and 15 cases in the disease group.

### 2.3 Immune cell infiltration analysis

The CIBERSORT method is a widely used method to evaluate immune cell types in the microenvironment. This method is based on the principle of support vector regression and performs deconvolution analysis on the expression matrix of immune cell subtypes. It contains 547 biomarkers that distinguish 22 human immune cell phenotypes, including T cells, B cells, plasma cells, and myeloid cell subsets. This study used the CIBERSORT algorithm to analyze patient data to infer the relative proportions of 22 types of immune infiltrating cells. CIBERSORT and each algorithm/tool used in this study and references are included in Supplementary Table S2.

### 2.4 WGCNA analysis

By constructing a weighted gene co-expression network, we can find co-expressed gene modules and explore the correlation between gene networks and diseases, as well as the core genes in the network. The WGCNA-R package was used to construct a co-expression network of all genes in the data set. The weighted adjacency matrix is converted into a topological overlap matrix (TOM) to estimate the network connectivity, and the hierarchical clustering method is used to construct the clustering tree structure for the TOM matrix. Different branches of the clustering tree represent different gene modules, and different colors represent different modules. Based on the weighted correlation coefficient of genes, genes are classified according to their expression patterns. Genes with similar patterns are grouped into one module, and all genes are divided into multiple modules according to their expression patterns.

### 2.5 Functional enrichment analysis

Genes were functionally annotated using the R package “ClusterProfiler” to comprehensively explore the functional correlation of these intersection genes. Gene Ontology (GO) and Kyoto Encyclopedia of Genes and Genomes (KEGG) were used to evaluate relevant functional categories. GO and KEGG enriched pathways with both *p*-value and *q*-value less than 0.05 were considered as significant categories.

### 2.6 Lasso regression and random forest feature selection

We use Lasso logistic regression and random forest algorithms for feature selection of diagnostic markers of diseases. The Lasso algorithm uses the “glmnet” package. Random forest is an ensemble learning algorithm based on decision trees. It uses sampling and replacement methods to select multiple samples from the sample set as the training set, and generates a decision tree using the sampled sample set. At each generated node, features are randomly selected without repetition, and these features are used to divide the sample set respectively, the best dividing features were found, and the prediction results were determined. This study used the random forest algorithm to evaluate the importance of features based on %IncMSE, and selects the top 10 features for subsequent analysis.

### 2.7 Gene set difference analysis (GSVA)

Gene set variation analysis (GSVA) is a non-parametric, unsupervised method for assessing transcriptome gene set enrichment. GSVA converts gene-level changes into pathway-level changes by comprehensively scoring the gene set of interest, and then determines the biological function of the sample. This study downloaded gene sets from the Molecular signatures database, and used the GSVA algorithm to comprehensively score each gene set to evaluate potential biological function changes in different samples.

### 2.8 GSEA pathway enrichment analysis

The differences in signaling pathways between high and low expression groups were further analyzed through GSEA. The background gene set is a version 7.0 annotated gene set downloaded from the MsigDB database. As an annotated gene set for subtype pathways, differential expression analysis was performed on pathways between subtypes, and a significantly enriched gene set (adjusted *p*-value less than 0.05) was classified. GSEA analysis is often used in studies that closely combine disease classification and biological significance.

### 2.9 Transcriptional regulation analysis of key genes

This study used the R package “RcisTarget” to predict transcription factors. All calculations performed by RcisTarget are based on motifs. The normalized enrichment score (NES) of a motif depends on the total number of motifs in the database. In addition to the motifs annotated by the source data, we also inferred further annotation files based on motif similarity and gene sequence. The first step in estimating the overexpression of each motif on a gene set is to calculate the area under the curve (AUC) for each motif-motif set pair. This was performed based on recovery curve calculations of the gene set against the ordering of the motifs. The NES of each motif is calculated based on the AUC distribution of all motifs in the gene set.

### 2.10 Genecards analysis

Genecard (https://www.genecards.org/) is a searchable, integrated human genome database that provides comprehensive, user-friendly information on all annotated and predicted human genes. The knowledge base automatically integrates gene-centric data from approximately 150 networked sources, including genomic, transcriptomic, proteomic, genetic, clinical, and functional information. Genecard database was used to search the keywords “preeclampsia” to obtain disease-related genes, and the co-expression of disease-related genes and key genes was analyzed.

### 2.11 GWAS analysis

The Gene Atlas database (http://geneatlas.roslin.ed.ac.uk/) is a large database that records associations between hundreds of traits and millions of variants using the UK Biobank cohort. These associations were calculated using 452,264 UK individuals from the UK Biobank database, covering a total of 778 phenotypes and 30 million loci.

### 2.12 CMap drug prediction

The Connectivity Map (CMap) is a gene expression profile database developed by Broad Institute based on intervention gene expression. It is mainly used to reveal the functional connections between small molecule compounds, genes and disease states, containing microarray data of 1,309 small molecule drugs before and after treatment of three human cell lines. There are various treatment conditions, including different drugs, different concentrations, different treatment times, etc. This study predicts targeted therapeutic drugs for diseases based on differentially expressed genes of the disease.

### 2.13 Western blot

Placental tissues were lysed in protein lysis buffer (#P0013B, Beyotime, China) containing 1% protease inhibitor (#B14002, Bimake, United States) and 1% phosphatase inhibitor (#B15001/B15002, Bimake, United States). After centrifugation at 12,000 g for 15 min at 4°C to remove tissue debris, the supernatant was collected as total protein. Protein concentration was determined using the BCA protein assay kit (#P0009, Beyotime, China). Protein samples were then mixed with sample buffer containing SDS (#1610747, Bio-Rad, United States), denatured at high temperature, and loaded onto polyacrylamide gels (#PG112, EpiZyme, China) for electrophoresis at a constant voltage of 100 V. Proteins from the SDS-PAGE gel were transferred onto PVDF membranes (#03010040001, Millipore, Germany) under constant voltage conditions at 100 V. The membrane was blocked with 5% skim milk for 1 h, washed with TBST, and then incubated overnight at 4°C with anti-NMUR1 antibody (1:1,000, #GTX115396, GeneTex, United States), anti-FNBP1L antibody (1:1,000, #GTX132563, GeneTex, United States), and anti-GAPDH antibody (1:1,000, #GB12002, Servicebio, China). The membrane was washed and then incubated with HRP-conjugated secondary antibodies (1:5,000, #SA00001, Proteintech, China) at room temperature for 1 h. Protein bands were detected using chemiluminescence detection reagent (#K-12045-D50, Advansta, United States) and captured with a ChemiDoc XRS+ system (Bio-Rad, United States). Gel lanes were analyzed with ImageJ, and statistical analysis was performed with GraphPad Prism 9.0.2.

### 2.14 Real time-quantitative polymerase chain reaction (qRT-PCR)

Tissues were homogenized in Trizol (#R401-01, Vamyaza, China) and total RNA was extracted using chloroform and precipitated with isopropanol. RNA was dissolved in RNase-free water (#R0022, Beyotime, China) after washing with 75% ethanol. The concentration of RNA was measured using ultraviolet spectroscopy (NanoDrop 2000, Thermo Fisher, United States). cDNA was synthesized using a reverse transcription kit (#HY-K0511A, MCE, United States). Gene expression was detected using SYBR Green dye (#HY-K0523, MCE, USA) and the Bio-Rad CFX Connect™ Real-Time System (#1855201, Bio-Rad, United States). PCR cycling conditions were set as follows: 95°C for 5 min, followed by 40 cycles of denaturation at 95°C for 10 s, annealing at 55°C for 20 s, and extension at 72°C for 10 s, final extension at 72°C for 30 s. GAPDH was used as an internal reference gene, and data analysis was performed using the 2^−ΔΔ^CT method. Statistical analysis was conducted using GraphPad Prism 9.0.2. The primer sequences were: Human-PP14571 (forward, GAT​CTG​CCT​TAG​GGA​GAA​GTC​TGA; reverse, AAA​GCA​AAC​AAC​TCT​TCT​CCC​CAA).

### 2.15 Immunofluorescence

The fixed placental tissue was sliced into 4-μm sections and placed on glass slides. After deparaffinization and dehydration, antigen retrieval was performed by exposing the sections to sodium citrate buffer at high temperature to restore antigen immunoreactivity. Blocking with goat serum was followed by overnight incubation with primary antibodies against NMUR1 (1:100, #GTX115396, GeneTex, United States) and CK7 (1:100, #22208-1-AP, Proteintech, China). Sections were then incubated with fluorescently labeled secondary antibodies (1:200, #SA00009, Proteintech) for 2 h in the dark, washed, and DAPI was added to stain the cell nuclei. After mounting, sections were observed under a microscope. ImageJ was used for localization and quantitative analysis of fluorescence intensity in placental villous tissue. Three independent experiments were conducted, with three random fields selected for statistical analysis from each sample in each experiment.

### 2.16 Statistical analysis

The differential gene screening conditions were *p*-value < 0.05 and |logFC| >0.585. All bioinformatics statistical analyses were performed in R language (version 4.2.2), with between-group comparisons conducted using the Wilcoxon rank-sum test; while all molecular experimental statistical analyses were performed in Graphpad Prism (version 9.0.2), conducted using the Student’s t-test. In this article, Test level was set at both sides *α* = 0.05, between-group differences with *p*-values < 0.05 were considered statistically significant, denoted as *p < 0.05, **p < 0.01, ***p < 0.001.

## 3 Results

**FIGURE 1:**
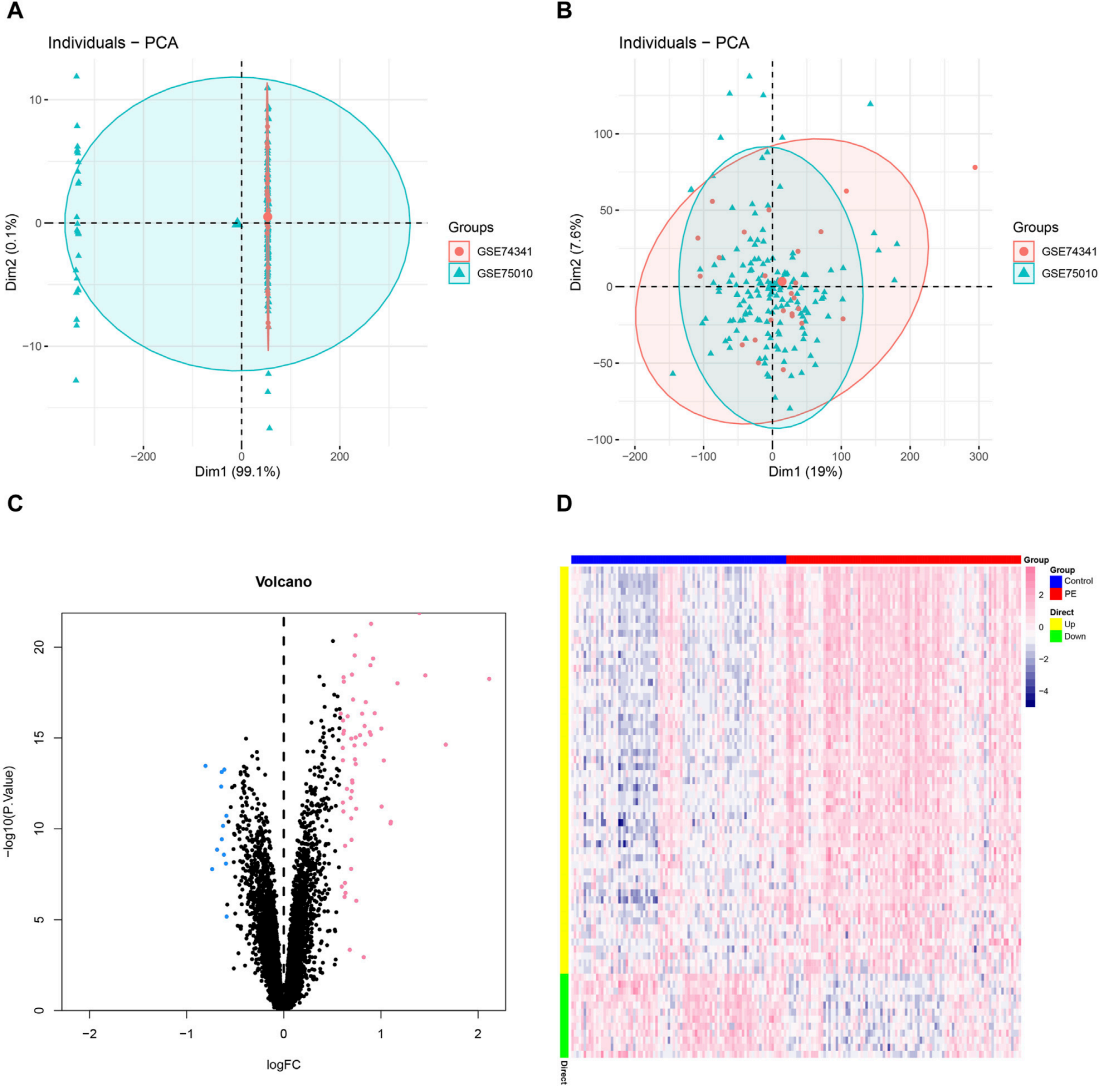
Batch effect analysis. **(A–B)** Batch effect before and after correction. **(C)** Volcano plot of differentially expressed genes, with pink indicating upregulation of differential expression and blue indicating downregulation of differential expression. **(D)** Heatmap of differential genes, blue indicates high expression and red indicates low expression.

We downloaded the data set related to GSE75010, GSE74341 and preeclampsia from the GEO database, with a total of expression profile data of 182 patients, including the control group (*n* = 87) and the disease group (*n* = 95). The SVA algorithm was used to correct the chip, and the PCA chart was used to show the batch situation before and after correction. The results showed that the inter-chip batch effect is reduced after SVA algorithm correction (Figures 1A,B). We used the limma package to calculate the differential genes between the control group and the disease group (Figures 1C,D). A total of 70 differential genes were screened out, including 58 upregulated genes, 12 downregulated genes.

**FIGURE 2:**
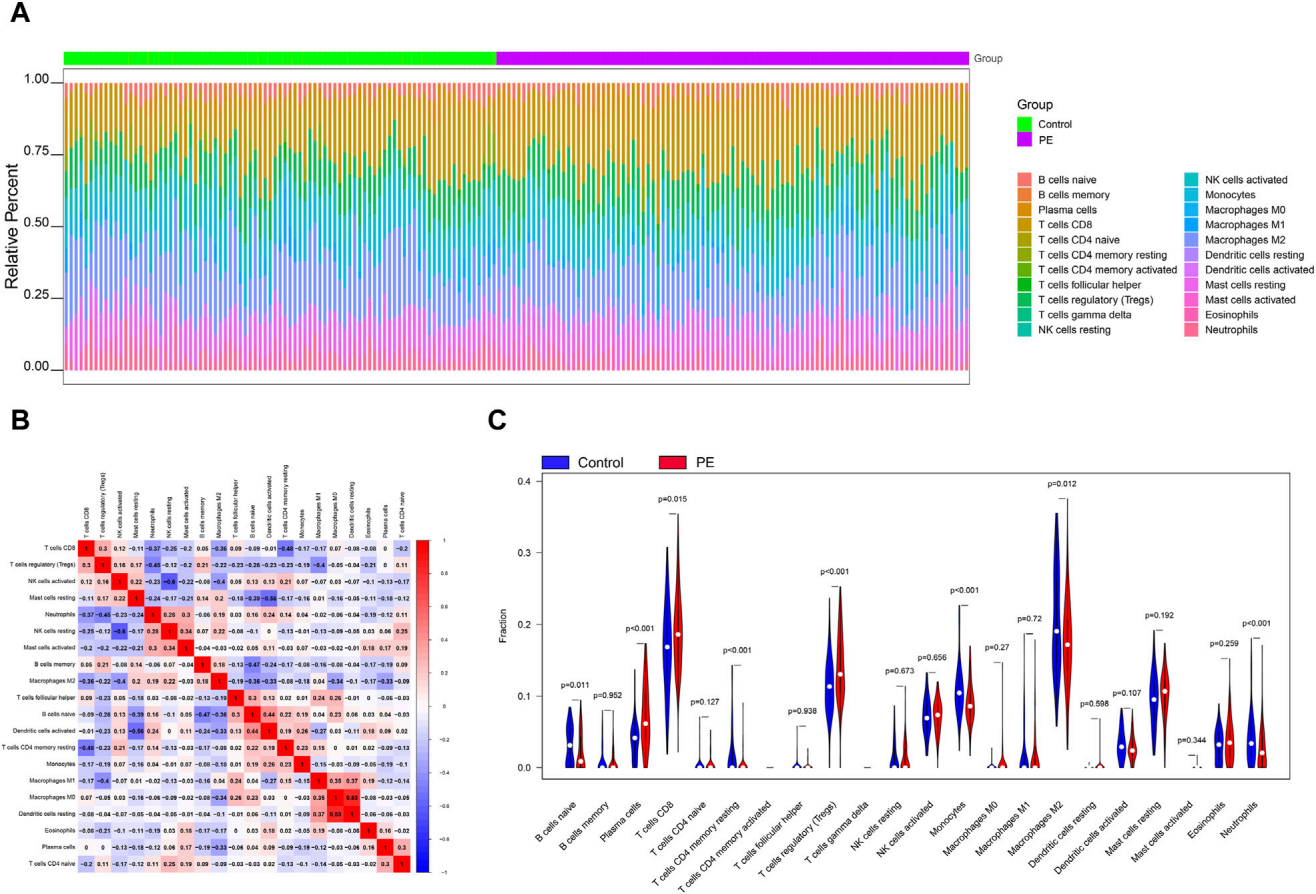
Immune infiltration analysis. **(A)** Relative percentages of 22 immune cell subsets. **(B)** Pearson correlation between 22 types of immune cells, blue indicates positive correlation, red indicates positive correlation. **(C)** Differences in immune cell content between control and disease samples.

The microenvironment is mainly composed of immune cells, extracellular matrix, various growth factors, inflammatory factors and special physical and chemical characteristics, which significantly affects the diagnosis of diseases and the sensitivity of clinical treatment. By analyzing the relationship between genes and immune infiltration in the preeclampsia data set, we further explored the potential molecular mechanisms by which key genes influence the progression of preeclampsia. The proportion of immune cell content in each patient and the correlation between immune cells are shown in the Figures 2A,B. In addition, the results showed a significant difference (*p* < 0.05) between the two groups of patients. Compared with normal pregnant women, macrophage M2 in PE patients decreased (Figure 2C).

**FIGURE 3:**
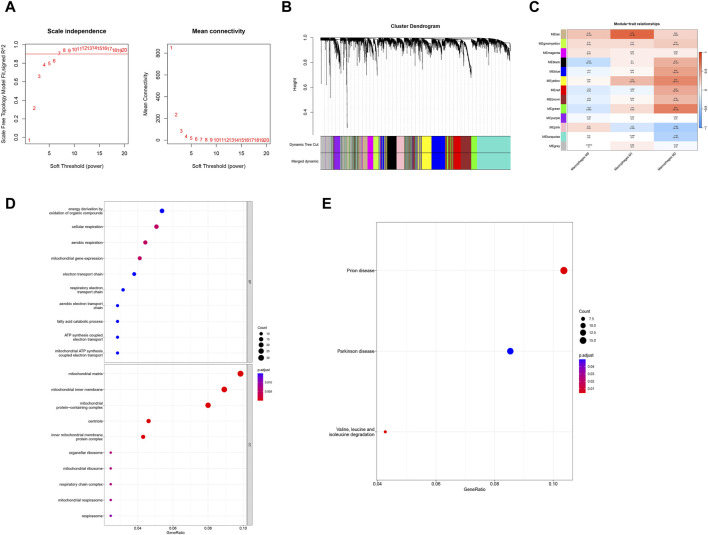
WGCNA and enrichment analysis. **(A)** Scale-free index and average connectivity for each soft threshold. **(B)** Dendrogram of gene clustering, with different colors representing different modules. **(C)** Heatmap of correlations between module signature genes, with blue indicating negative correlation and red indicating positive correlation. **(D, E)** GO-KEGG enrichment analysis based on ClusterProfiler.

In order to identify the key genes in the preeclampsia cohort, we further constructed the WGCNA network based on expression profile data combined with macrophages. In the data set, the soft threshold *β* is set to 7 (Figure 3A), and then the gene module is detected based on the tom matrix. A total of 13 gene modules were detected in this analysis (Figures 3B,C), respectively turquoise (1072), black (335), pink (232), red (355), blue (541), green (367), gray (564), brown (481), greenyellow (159), magenta (206), purple (170), tan (149), yellow (369), among which the green module has the highest correlation with Macrophages M2 (cor = 0.61, p = 5e−20). We conducted pathway analysis on 367 module genes of the green module. The GO enrichment analysis results showed that the cellular respiration, mitochondrial gene expression, aerobic respiration and other pathways were mainly enriched (Figure 3D); the KEGG enrichment analysis results showed that the mainly enriched pathways of Valine, leucine and isoleucine degradation are integrated (Figure 3E).

**FIGURE 4:**
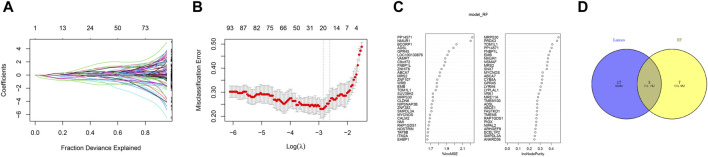
Random Forest. **(A)** Distribution of LASSO coefficients of intersection genes and gene combinations at minimum lambda values. **(B)** Ten-fold cross-validation of tuning parameter selection in the LASSO model to determine the minimum lambda value. **(C)** Display of random forest feature genes. **(D)** Venn diagram of LASSO and RF genes.

**FIGURE 5:**
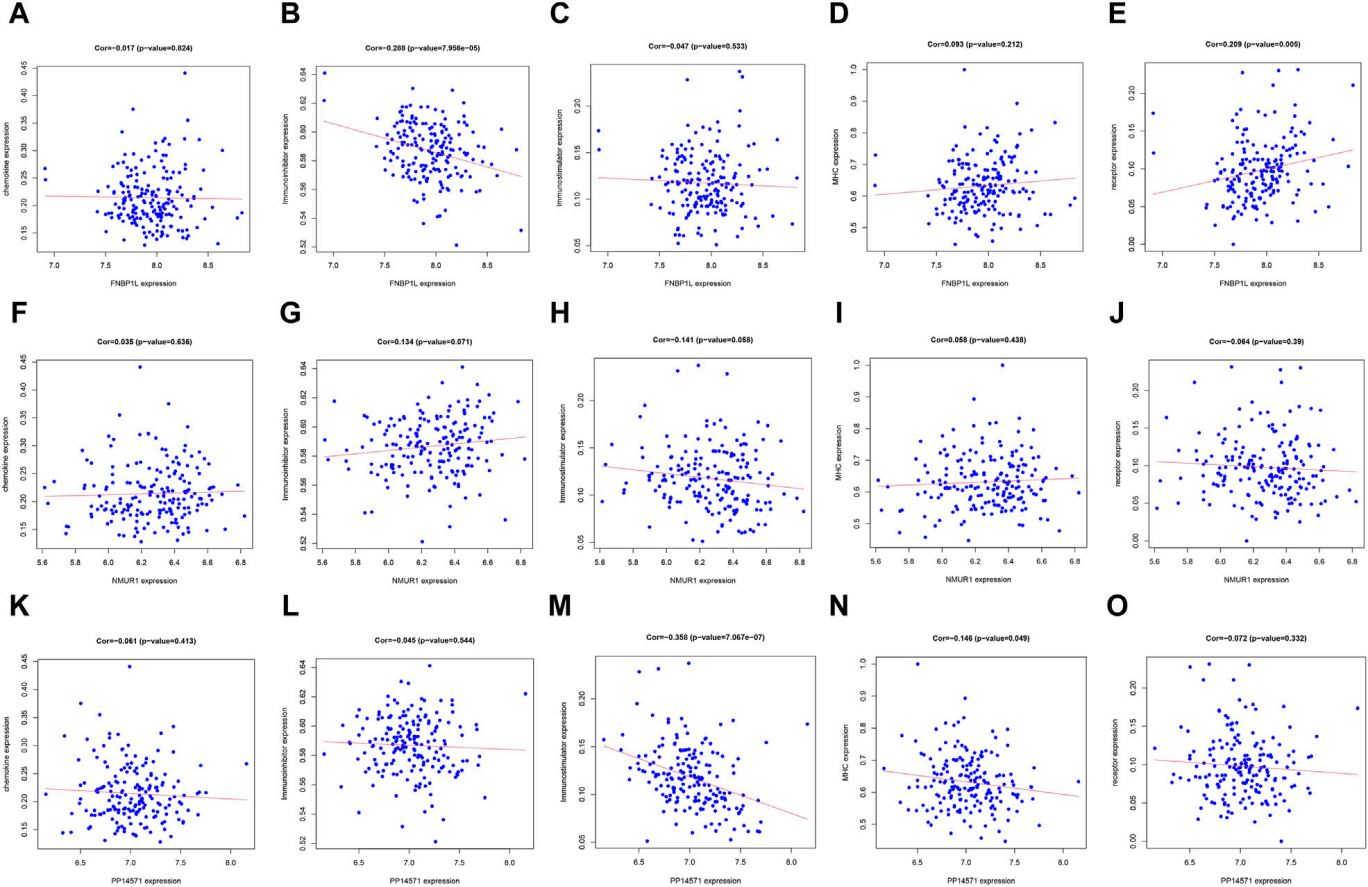
Relationship between key genes and immune factors. **(A–O)** Correlation of FNBP1L, NMUR1 and PP14571 genes with chemokine, Immunoinhibitor, Immunostimulator, MHC and receptor.

In order to further identify the key genes that affect preeclampsia, we used green module genes to screen out characteristic genes in preeclampsia through lasso regression and random forest. The results showed that Lasso regression identified a total of 20 genes as characteristic genes for preeclampsia (Figures 4A,B). We utilized the random forest algorithm to rank the importance of genes associated with preeclampsia, selecting the top 10 genes (Figure 4C). These genes intersected with the characteristic genes identified by the Lasso regression algorithm, and a total of three intersection genes were screened out (Figure 4D). These three genes, FNBP1L, NMUR1, and PP14571, will be the key focus for our subsequent research. In addition, we analyzed the correlation between three key genes and different immune factors from the TISIDB database, including immunosuppressive factors, immunostimulatory factors, chemokines, and receptors. These analyzes indicate that key genes are closely related to the level of immune cell infiltration and play an important role in the immune microenvironment (Figure 5). Based on the results, FNBP1L shows a negative correlation with immunosuppressive factors, while NMUR1 exhibits a positive correlation with immunosuppressive factors. Additionally, PP14571 is positively associated with immune stimulatory factors and MHC.

**FIGURE 6:**
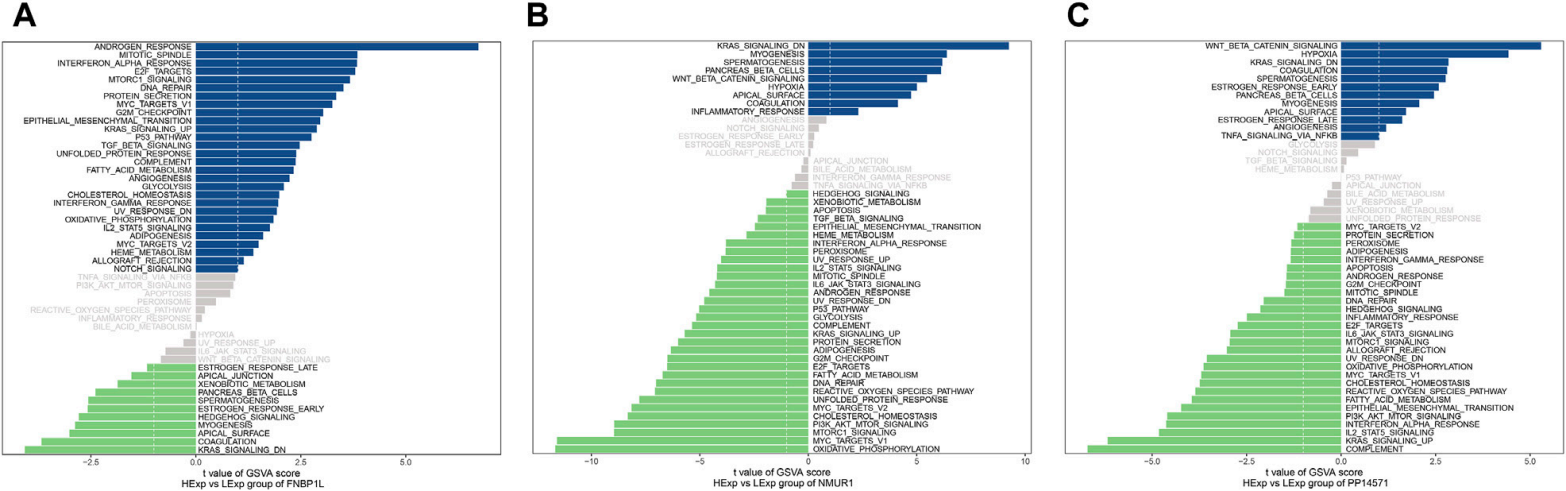
GSVA analysis of key genes. **(A–C)** GSVA analysis of FNBP1L, NMUR1 and PP14571 genes. Blue indicates the signaling pathways involved in high expression genes, while green indicates the signaling pathways involved in low expression genes. The background gene set is hallmark.

**FIGURE 7:**
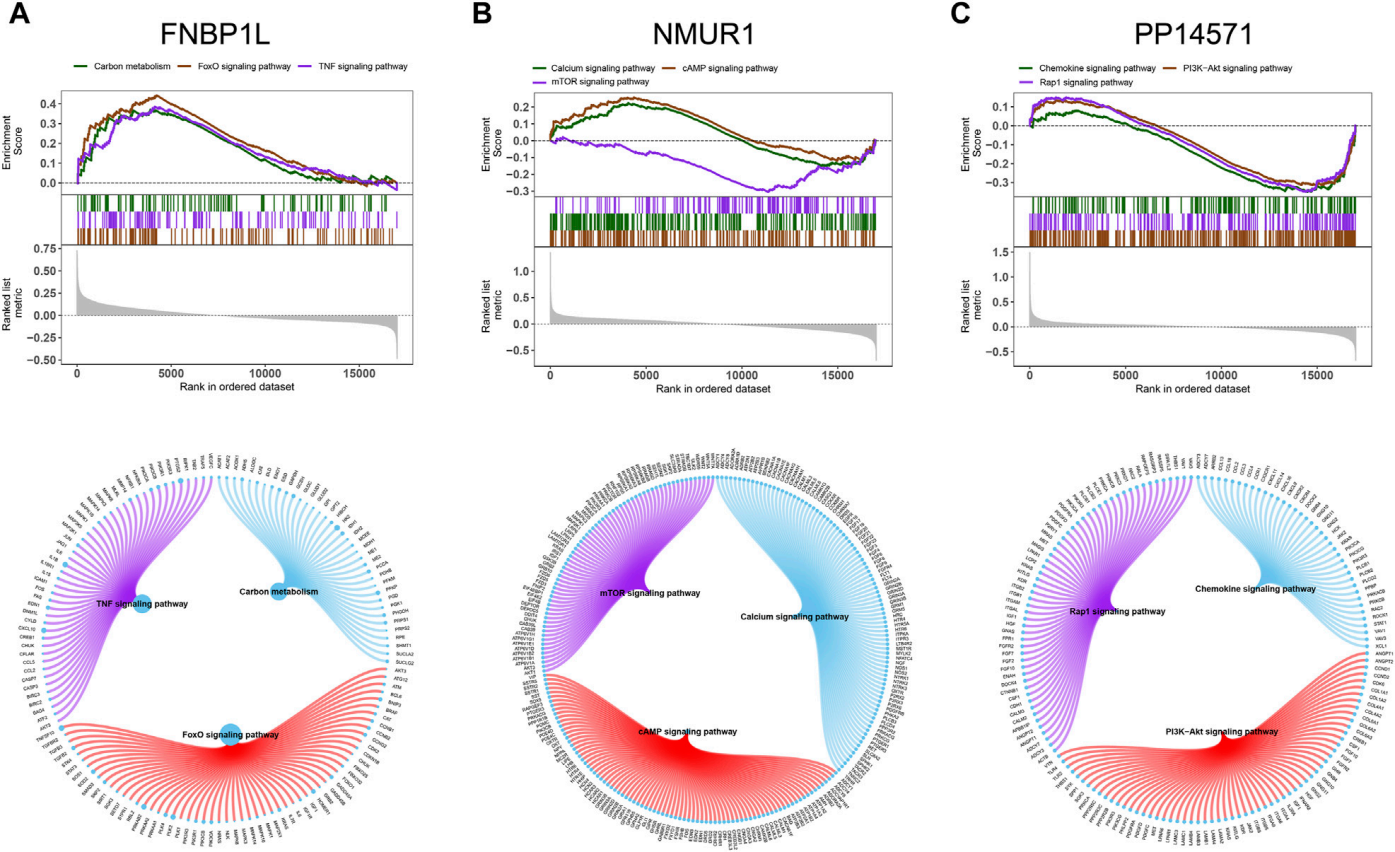
GSEA analysis of key genes. **(A–C)** The KEGG signaling pathway involved in FNBP1L, NMUR1 and PP14571 genes, as well as pathway regulation and involved genes.

Next, we investigated specific signaling pathways rich in key genes and explored the potential molecular mechanisms by which key genes affect the progression of preeclampsia. The GSVA results showed that the pathways enriched by FNBP1L include ANDROGEN_RESPONSE, MTORC1_SIGNALING, KRAS_SIGNALING_UP and other pathways (Figure 6A); The pathways enriched by NMUR1 include KRAS_SIGNALING_DN, WNT_BETA_CATENIN_SIGNALING and other pathways (Figure 6B); the pathways enriched by PP14571 include WNT_BETA_CATENIN_SIGNALING, KRAS_SIGNALING_DN and other pathways (Figure 6C). In addition, GSEA results showed that pathways enriched by FNBP1L include Carbon metabolism, FoxO signaling pathway, TNF signaling pathway and other pathways (Figure 7A); pathways enriched by NMUR1 include Calcium signaling pathway, cAMP signaling pathway, mTOR signaling pathway and other pathways (Figure 7B); the pathways enriched by PP14571 include Chemokine signaling pathway, PI3K-Akt signaling pathway, Rap1 signaling pathway, and other pathways (Figure 7C).

**FIGURE 8:**
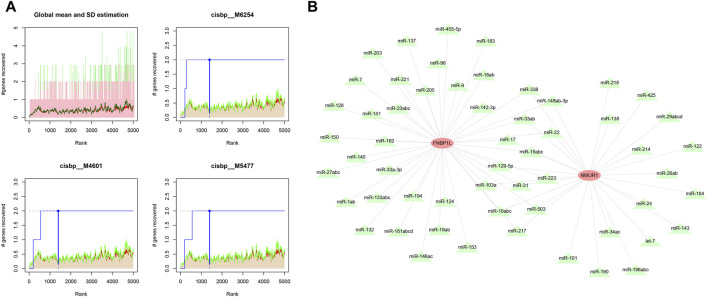
Motif transcriptional regulation and ceRNA analysis of key genes. **(A)** Four motifs with higher AUC. The red line in the Figure is the average recovery curve of each motif, the green line is the average + standard deviation, and the blue line is the recovery curve of the current motif. The maximum distance point between the current motif and the green curve (mean + SD). **(B)** miRNA network of key genes, light red represents mRNA and light green represents miRNA.

We used these three key genes as the gene set for further transcription factor prediction and found that they are regulated by common mechanisms such as multiple transcription factors. Therefore, the cumulative recovery curve was used for enrichment analysis of these transcription factors. Motif-TF annotation, and the selection analysis results of important genes showed that the Motif with the highest normalized enrichment score (NES: 9.6) is cisbp__M6254. We demonstrated all enriched motifs and corresponding transcription factors of key genes (Figure 8A). In addition, we performed reverse prediction of key genes through the miRcode database and obtained 56 miRNAs, a total of 68 pairs of mRNA-miRNA relationships, and visualized them using cytoscape (Figure 8B).

**FIGURE 9:**
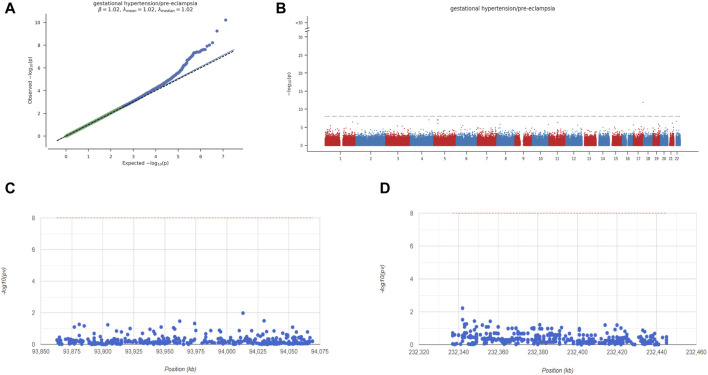
Key overview of GWAS analysis. **(A)** Q-Q plot shows that significantly associated SNP sites can be identified in GWAS data. **(B)** Manhattan plot representing meta-GWAS results. **(C-D)** Chromosomal locations of 2 key genes.

Next, we analyzed the GWAS data of preeclampsia to confirm the causative regions of 3 key genes in the disease. The Q-Q diagram showed significant single nucleotide polymorphism (SNP) loci identified by GWAS data related to the disease (Figure 9). Through precise positioning of GWAS data, key SNP sites distributed in enriched areas were described. It also showed the SNP pathogenic regions corresponding to FNBP1L and NMUR1. FNBP1L is located in the pathogenic region of chromosome 1, and NMUR1 is located in the pathogenic region of chromosome 2. The significant SNP sites corresponding to the two genes are shown in the table (GWAS data.xlsx).

**FIGURE 10:**
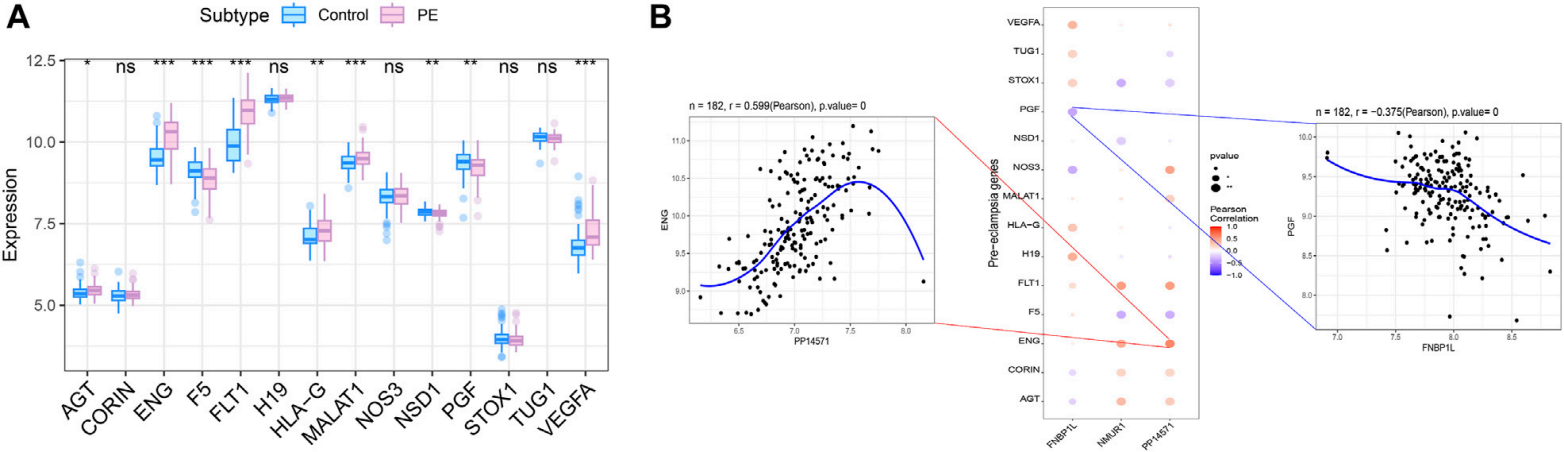
Correlation analysis between key genes and disease progression genes. **(A)** Differences in the expression of disease-regulated genes, with blue indicating control patients and pink indicating disease patients. **(B)** Pearson correlation analysis of key genes and disease genes. Blue indicates negative correlation and red indicates positive correlation.

We obtained preeclampsia-related disease genes through the GeneCards database. Analyzing the expression differences of disease genes between groups, it was found that the expression of AGT, ENG, F5, FLT1, HLA-G, MALAT1, NSD1, PGF, VEGFA and other genes were different between the two groups of patients (Figure 10A). We analyzed the expression levels of three key genes and the expression levels of the top 20 genes with Relevance score, and found that the expression levels of these three genes were significantly correlated with the expression levels of multiple disease-related genes (Figure 10B), with PP14571 and ENG showing a significant positive correlation (Pearson r = 0.599), and FNBP1L showing a significant negative correlation with PGF (Pearson r = −0.375).

**FIGURE 11:**
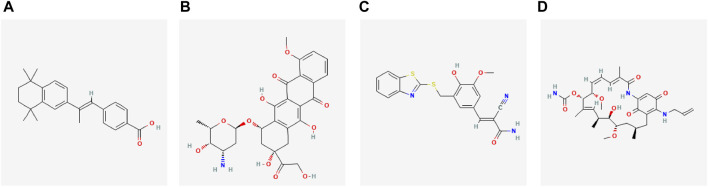
Potential therapeutic drugs for diseases. **(A–D)** 2D structure diagrams of these four drugs, namely Ttnpb, Doxorubicin, Tyrphostin AG 825, and Tanespimycin.

We used the Connectivity Map database to predict drug targets for differential genes in preeclampsia. The results showed that the expression profiles perturbed by drugs such as Ttnpb, Doxorubicin, Tyrphostin AG 825, Tanespimycin, etc., were negatively correlated with the expression profiles perturbed by diseases, and the drugs can alleviate or even reverse the disease state (Figure 11).

**FIGURE 12:**
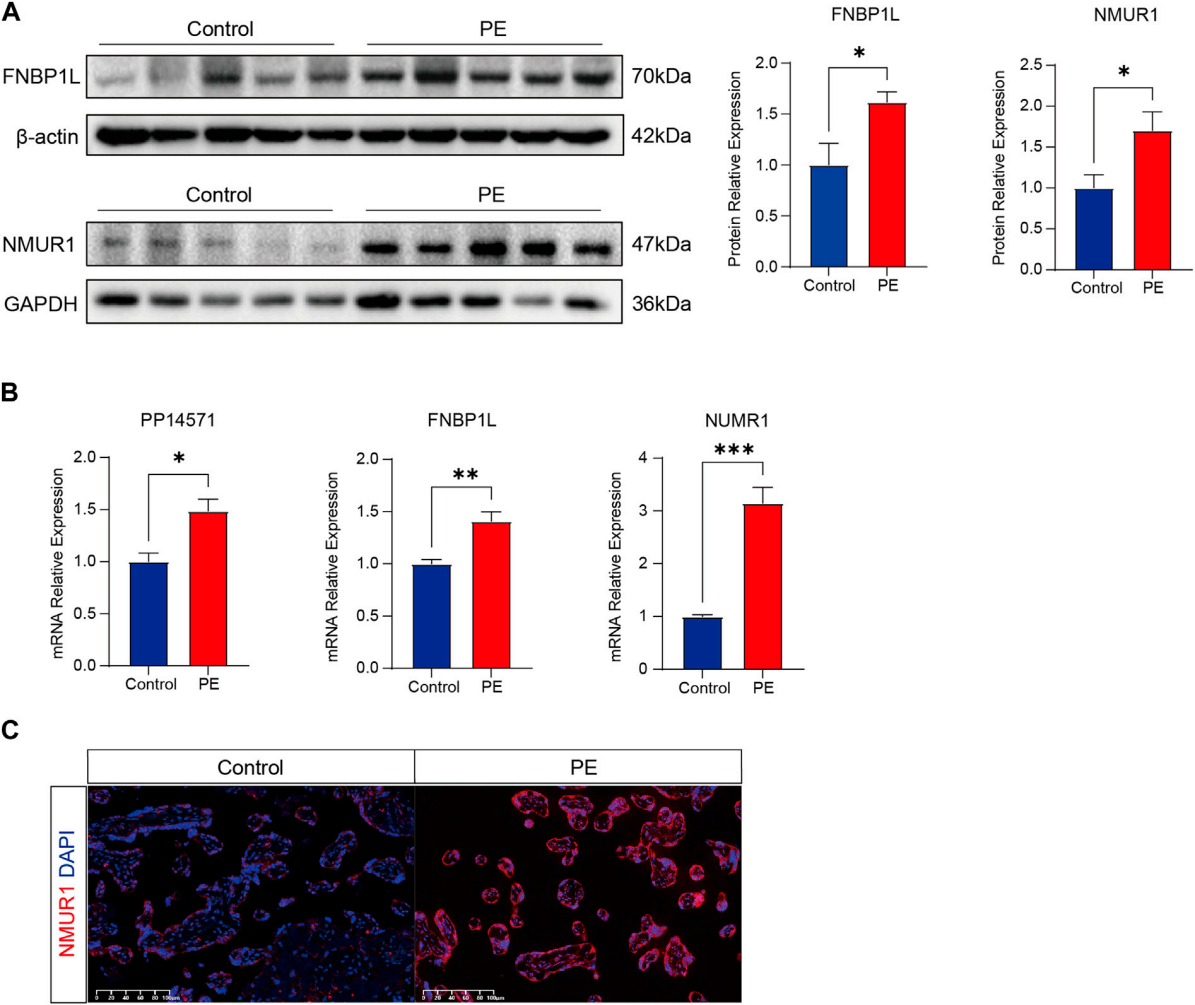
Verifying the abnormal expression of FNBP1L and NMUR1 in PE placenta. **(A)** Western blot analysis of NMUR1 and FNBP1L protein expression levels in human placental tissues of normal and PE patients. **(B)** The transcription levels of NMUR1, FNBP1L and PP14571 in human placental tissues of normal and PE patients were determined by RT-qPCR. **(C)** Immunofluorescence staining of NMUR1 in human placental villi from normal and PE patients.

Finally, to further confirm the association of FNBP1L, NMUR1, and PP14571 with immune infiltration in PE patients, we conducted tissue gene expression and protein level validation of these three key genes in human placental tissues. Western Blot results showed that the expression of FNBP1L and NMUR1 in placental tissues of PE patients was higher than that of normal pregnant women (Figure 12A). Similarly, qRT-PCR validated the gene transcription levels of FNBP1L, NMUR1, and PP14571 (Figure 12B). Additionally, immunofluorescence revealed NMUR1 localization in the villous trophoblast layer of the placenta (Figure 12C), indicating its involvement in the maternal-fetal interface immune microenvironment of PE patients.

Changes in the expression levels of these genes may directly or indirectly affect the immune infiltration of M2 macrophages, thereby influencing the occurrence and development of PE. The expression level of the FNBP1L gene is significantly upregulated in PE patients, which may be related to abnormal immune responses in these individuals. As a cytoskeleton-related protein, FNBP1L plays a crucial role in maintaining cell structure and function, and changes in its expression level may directly affect the activity and function of immune cells. Secondly, NMUR1 encodes the neuromedin U receptor, which is closely related to the regulation of the neuroendocrine system. In PE patients, alterations in NMUR1 expression may affect the neuroendocrine system’s regulation of the immune system, further influencing the disease progression of PE. Although the specific function of PP14571 is not yet fully understood, its potential role in immune regulation cannot be ignored. Changes in PP14571 expression may affect the differentiation, activation, and function of immune cells, thereby participating in the pathological process of PE. Therefore, further studies on the functions of these genes and their relationship with PE are expected to provide new ideas and methods for the diagnosis and treatment of PE.

## 4 Discussion

PE is a common hypertensive disorder during pregnancy, affecting 4.6% of pregnancies worldwide (Abalos et al., 2013), causing severe harm to maternal and fetal health. In China, two-thirds of cases of preeclampsia are classified as severe PE, and the perinatal mortality rate is three times that of other countries (Yang et al., 2021). The risk factors associated with preeclampsia include age, multiple pregnancies, and obesity, which are one of the most important risk factors for PE in China. Among various contributing factors to PE, maternal genetics play a more substantial role than fetal genetics, with a heritability estimate of approximately 55% (Steinthorsdottir et al., 2020). Genome-wide association studies (GWAS) have identified sequence variants in the maternal genome associated with PE. However, the precise pathogenic mechanisms of PE remain unclear. Therefore, clinical management often focuses on symptom relief through antihypertensive, antispasmodic, and sedative measures. The only definitive solution is to terminate pregnancy and deliver the placenta early, which can alleviate symptoms to some extent, reduce complications, and lower mortality rates. However, terminating pregnancy does not eliminate the impact of PE entirely.

Pregnancy is a semi-allogeneic transplant process, involving macrophages and natural killer cells in regulating trophoblast invasion, vascular development, and spiral artery remodeling. Immune mechanisms play a vital role throughout the process, from embryo implantation and placental formation to delivery. The placenta acts as a barrier for fetal-maternal exchange and plays a crucial role in fetal development. Immune infiltration at the maternal-fetal interface, including macrophages, natural killer cells, and T lymphocytes, is often observed in the decidua and placenta associated with PE (Bu et al., 2022). This complex and dynamic ecosystem is similar to a finely tuned balance that requires the participation of the maternal immune system and various adaptations during placental development. In the early stages of trophoblast invasion and spiral artery remodeling, the uterine decidua contains numerous immune cells, allowing maternal immune tolerance to ensure proper placental implantation and trophoblast invasion, depending on the successful function of the maternal immune system (Miller et al., 2022). When immune imbalance occurs at the maternal-fetal interface, pathological inflammatory responses develop, affecting trophoblast invasion, leading to placental hemorrhage, systemic inflammation, endothelial dysfunction, increased oxidative stress, and vascular damage, all of which contribute to the pathophysiology of PE (Sultana et al., 2017). Some studies have attempted to predict the occurrence of PE by measuring circulating biomarkers related to maternal vascular system or endothelial dysfunction (MacDonald et al., 2022), but the predictive accuracy has been limited. Therefore, identifying key genes related to preeclampsia and managing and intervening in PE through these genes has significant clinical significance.

We conducted an in-depth analysis of immune cell infiltration in placental tissues from PE and non-PE groups. Using the CIBERSORT algorithm, we found that M2-type macrophages were significantly lower in the PE group compared to the non-PE group, while Tregs were significantly lower in the PE group. Due to the crucial role of macrophages in the microenvironment of immune infiltration during this process, we analyzed the differential gene expression profiles in placental tissues of preeclampsia and non-PE groups, and constructed a weighted gene co-expression network analysis (WGCNA) network, further conducting enrichment analysis of differentially expressed genes (DEGs). In this study, we observed significant differences in the expression levels of FNBP1L, NMUR1, and PP14571 genes in patients with preeclampsia. Through analyzing the relationship between key genes and immune molecules, we observed a negative correlation between FNBP1L and immunosuppressive molecules, while NMUR1 showed a positive correlation with immunosuppressive molecules. In order to prevent maternal immune rejection of the fetus, M2 macrophages typically exhibit anti-inflammatory effects in the maternal-fetal immune microenvironment. Previous studies have reported a decrease in M2 macrophages in the placenta of PE patients, consistent with our immunoinfiltration analysis results. Through specific experimental validation, we found that FNBP1L, which negatively correlates with immunosuppressive molecules, is significantly elevated in PE patients, while NMUR1 shows the opposite trend. Their respective levels of increase and decrease are similar to the decrease of M2 macrophages in PE patients. We believe that these genes play a crucial regulatory role in the maternal infant immune microenvironment of PE patients and are closely related to the immune infiltration of M2 macrophages. Furthermore, through GWAS databases, we identified three key genes in the disease’s pathogenic regions.

The FNBP1L gene’s exons 1-7 are located within the human genome sequence AL512651.13, while exons 7-15 are within AL109613.11, involved in regulating cell shape, polarity, motility, and signaling (Katoh and Katoh, 2004). Previous studies have suggested that the FNBP1L gene, encoding formin-binding protein 1-like protein, is related to neurodevelopment in adults and children (Benyamin et al., 2014). It exhibits significant alterations in the Alzheimer Disease (AD) patient’s genome and may play a role in the neuroplasticity-related pathology of AD (Prokopenko et al., 2021). Additionally, tonsil-derived mesenchymal stem cells (MSCs) can differentiate into skeletal muscle cells and treat muscle-related diseases. In myogenic differentiation, key factors related to macrophage-associated autophagic signaling pathways are upregulated (Park et al., 2017). The interaction between the HR1 domain of FNBP1L and human Atg3 protein and its membrane binding are necessary for autophagic biochemical processes. However, except for the tonsils, the placenta, amniotic fluid, and umbilical cord blood can all be isolated to extract MSCs. According to the TISDB database, FNBP1L is negatively correlated with immunosuppressive factors and positively correlated with receptors. Currently, there is no direct research on the relationship between FNBP1L and PE, but FNBP1R may regulate the invasion of placental trophoblast cells in the maternal fetal immune environment during placental development through autophagy, which is a topic worth further exploration.

During early embryo implantation, both the trophoblast and outer cell layers secrete various growth factors and cytokines to regulate trophoblast differentiation and invasion at the maternal-fetal interface. It has been suggested that inadequate trophoblast infiltration leading to impaired uterine spiral artery remodeling may be crucial in the early stages of PE development. The upregulation of FNBP1L expression promotes human trophoblast invasion and plays a significant role in early embryo implantation (Wang et al., 2022).

Neuromedin (NUM) is a highly conserved neuropeptide in mammals (Lin et al., 2015), acting through its peripheral G protein-coupled receptor NMUR1. It was initially discovered to induce powerful uterine contractions in the rat uterus (Malendowicz and Rucinski, 2021). In humans, the NMUR1 gene is located on chromosome 2 and comprises three exons and two introns. It transduces NUM signals to regulate smooth muscle contraction and blood pressure control (Lin et al., 2015). NMUR1 is known to be expressed in all types of macrophages (Przygodzka et al., 2022), and research has confirmed that NMU activates macrophages through NMUR1, significantly phosphorylating the ERK1/2 pathway, and exerting its effects. Therefore, we speculated that the endothelial dysfunction characteristic of PE patients with high NMUR1 expression may result from NMU secreted by endothelial cells, activating NMUR1-positive endothelial cells and infiltrating macrophages. Hence, NMUR1 would serve as a potential biomarker for the development of PE.

In our study, the expression of PP14571 in PE patients was significantly negatively correlated with MHC and immune stimulating molecules. Although there have been no reports on the functional role of PP14571 in biological processes and cellular activities to date, this lncRNA, as a non-coding RNA, can still play a role in various key regulatory processes, such as chromatin silencing, chromatin modification, transcriptional interference, and activation (Ballantyne et al., 2016). One of the prominent features of PE is inadequate trophoblast invasion, and genes or non-coding RNAs with abnormal expression primarily play a role in regulating trophoblast cell proliferation, apoptosis, and invasion. Additionally, a retrospective analysis found differential expression of lncRNA PP14571 in the prognosis model of breast cancer patients (Sun et al., 2021). Trophoblast cells exhibit regulated invasive behavior during early pregnancy, facilitating their entry into the uterine wall, establishing the placenta, ensuring the fetus receives sufficient nutrients and oxygen. Meanwhile, lncRNAs can act independently or in conjunction with miRNAs, affecting downstream target genes, regulating trophoblast cell invasion, inflammatory responses, and influencing uterine spiral artery remodeling. For example, upregulation of lncRNA XIST in trophoblast cells HTR8/Sveno can inhibit cell proliferation and invasion and increase apoptosis by interacting with miRNA-340-5p/KCNJ16 during myogenic differentiation (Guo et al., 2022). Overexpression of miRNA-340-5p or lncRNA NEAT1 can mitigate these effects by mediating the upregulation of miRNA-411 (Fan et al., 2021), slowing the progression of PE. Therefore, lncRNAs are also significant contributors to trophoblast cell function, making them essential factors in the development of PE. This finding provided support for our analysis.

Subsequently, we used GSVA and GSEA to enrich the signaling pathways of key genes and predicted the target miRNAs of FNBP1L, NMUR1, and PP14571 to explore potential RNA regulatory pathways that control the progression of PE. However, further validation is needed to confirm our findings (Richardson et al., 2010).

Finally, The drugs we predicted by the Connectivity Map database such as Ttnpb, Doxorubicin, Tyrphostin AG 825, and Tanespimycin have not been applied in obstetrics yet, they have shown anticancer effects or inhibited cell proliferation in other diseases and may be effective in improving or even reversing the condition of PE, particularly in facilitating uterine spiral artery remodeling. Ttnpb is a cancer treatment for HER2-positive breast cancer and is also an effective retinoic acid receptor (RAR) agonist, which has been shown to convert mouse fibroblasts into hair follicle stem cells (DPC-LC) (Ma et al., 2022). Retinoic acid-like compounds play various biological activities at different stages of development, critical for normal embryonic development and subsequent cell proliferation, differentiation, and apoptosis (De Luca, 1991), mediated by RAR. Research has shown that the RAR agonist Ttnpb can promote ATP-binding cassette transporter A1 (ABCA1) mRNA and protein production in macrophages, facilitating cholesterol efflux (Costet et al., 2003). By modulating these pathways, it may be able to reduce the severity of symptoms or even prevent the onset of the disease, potentially improving outcomes for PE mothers and their fetuses.

Doxorubicin (Hydroxydaunorubicin) is a cytotoxic anthracycline antibiotic used as a chemotherapeutic agent for triple-negative breast cancer, killing cancer cells by inhibiting DNA synthesis and replication. While its use in obstetrics is still in the early stages, the translational impact of Doxorubicin could be significant if it is found to be effective and safe in clinical trials.

Tyrphostin AG 825 is a selective, ATP-competitive ErbB2 inhibitor that inhibits tyrosine phosphorylation, as a potential anti-Alzheimer’s disease drug, it has been confirmed to have effects on anticancer, accelerating neutrophil apoptosis. It may have a role in regulating immune responses and vascular function in preeclampsia, and the ability to modulate these crucial biological processes potentially leading to improvements in patient outcomes.

Tanespimycin depletes intracellular STK38/NDR1, reducing STK38 kinase activity, downregulating basal phosphorylation of AMPK, and acetyl-CoA carboxylase. In addition, as an HSP90 inhibitor, Tanespimycin induces tumor cell apoptosis and autophagy and has been applied in the treatment of refractory/relapsed multiple myeloma (Richardson et al., 2010). In gynecology, it has been used in combination with other drugs for HER2-positive metastatic breast cancer patients, significantly improving their prognosis (Modi et al., 2011). Given the association of preeclampsia with abnormalities in immune regulation, vascular function, and cellular stress responses, the known mechanisms of Tanespimycin may potentially play a role in the treatment of preeclampsia. Specifically, its ability to downregulate the basal phosphorylation of AMPK and acetyl-CoA carboxylase may positively impact the regulation of cellular energy metabolism and stress responses. The combined effects of these mechanisms may assist in alleviating the symptoms of PE and improving the prognosis for both mother and child. In summary, although the drugs we predicted have not yet been used for pregnancy related hypertension, they have shown anticancer effects or inhibited cell proliferation in other diseases. They may be effective in improving or even reversing the condition of PE, particularly in facilitating uterine spiral artery remodeling.

It is important to note that the translation of these preclinical findings into clinical practice requires rigorous testing in clinical trials. Safety, efficacy, and dosing need to be carefully evaluated to ensure that these compounds are suitable for use in pregnant women. Additionally, the potential side effects and long-term outcomes of these drugs must also be thoroughly investigated.

In conclusion, the potential translational impact of Ttnpb, Doxorubicin, Tyrphostin AG 825, and Tanespimycin for preeclampsia treatment is significant. However, further research and clinical trials are necessary to validate their therapeutic potential and ensure their safety and effectiveness in clinical practice.

Our study has some limitations. As a retrospective study in public databases, the available data meeting inclusion criteria are limited, and all analyzed data are sourced from public databases. Therefore, we use additional experimental evidence to confirm our findings. In this study, we identified three key genes, FNBP1L, NMUR1, and PP14571, which regulate immune cell infiltration levels in PE macrophages. We established potential pathways that may contribute to studying the molecular mechanisms of preeclampsia and developing therapeutic targets. Through the use of established protein and ligand structure databases based on key genes, we predicted drugs that can alleviate or even reverse this disease state, and obtained the predicted binding scores and application prospects of these drugs. Finally, we discussed the relevance between preeclampsia and the immune system. We hope that the above findings will assist in future in-depth research and contribute to the goal of improving the prognosis of PE patients.

## Data Availability

The datasets presented in this study can be found in online repositories. The names of the repository/repositories and accession number(s) can be found in the article/Supplementary Material.
