# Supplementary material for: Mechanistic study of pre-eclampsia and macrophage-associated molecular networks: bioinformatics insights from multiple datasets
Source: Front Genet. 2024 May 23;15:1376971. doi: 10.3389/fgene.2024.1376971 (PMC11153808; doi:10.3389/fgene.2024.1376971)
Supplement: Supplementary file 1 [file Table1.pdf]

Supplementary Material

Supplementary Table1.The comprehensive description of the datasets

GEO ID	GSE75010	GSE74341
Platform	[HuGene-1_0-st] Affymetrix Human Gene 1.0 ST Array [transcript (gene) version]	Agilent-039494 SurePrint G3 Human GE v2 8x60K Microarray 039381 (Feature Number version)
PE	80	15
Controls	77	10
Total Samples	157	25
PE definition	Preeclampsia (PE) is a complex, heterogeneous disorder of pregnancy, demonstrating considerable variability in observed maternal symptoms and fetal outcomes.	Not mentioned
Tissue	placental	placental

Supplementary Table2.The references for the CIBERSORT algorithm and for each algorithm or tool utilized in this study

Algorithm/Tool	Usage	Reference
Combat	The sva package for removing batch effects and other unwanted variation in high-throughput experiments.	ACOG Practice Bulletin No. 202: Gestational Hypertension and Preeclampsia. Obstet Gynecol 133(1), 1. doi: 10.1097/aog.0000000000003018.
Limma	Limma powers differential expression analyses for RNA-sequencing and microarray studies.	Ritchie, M.E., Phipson, B., Wu, D., Hu, Y., Law, C.W., Shi, W., et al. (2015). limma powers differential expression analyses for RNA-sequencing and microarray studies. Nucleic Acids Res 43(7), e47. doi: 10.1093/nar/gkv007.
Clusterprofiler	An R package for comparing biological themes among gene clusters.	Yu, G., Wang, L.G., Han, Y., and He, Q.Y. (2012). clusterProfiler: an R package for comparing biological themes among gene clusters. Omics 16(5), 284-287. doi: 10.1089/omi.2011.0118.
WGCNA	An R package for weighted correlation network analysis.	
Glmnet	Regularization Paths for Generalized Linear Models via Coordinate Descent.	Yu, G., Wang, L.G., Han, Y., and He, Q.Y. (2012). clusterProfiler: an R package for comparing biological themes among gene clusters. Omics 16(5), 284-287. doi: 10.1089/omi.2011.0118.
The Connectivity Map	using gene-expression signatures to connect small molecules, genes, and disease.	Lamb, J., Crawford, E.D., Peck, D., Modell, J.W., Blat, I.C., Wrobel, M.J., et al. (2006). The Connectivity Map: using gene-expression signatures to connect small molecules, genes, and disease. Science 313(5795), 1929-1935. doi: 10.1126/science.1132939.
CIBERSORT	Profiling Tumor Infiltrating Immune Cells with CIBERSORT.	Chen, B., Khodadoust, M.S., Liu, C.L., Newman, A.M., and Alizadeh, A.A. (2018). Profiling Tumor Infiltrating Immune Cells with CIBERSORT. Methods Mol Biol 1711, 243-259. doi: 10.1007/978-1-4939-7493-1_12.
Motif	SCENIC: single-cell regulatory network inference and clustering.	Aibar, S., González-Blas, C.B., Moerman, T., Huynh-Thu, V.A., Imrichova, H., Hulselmans, G., et al. (2017). SCENIC: single-cell regulatory network inference and clustering. Nat Methods 14(11), 1083-1086. doi: 10.1038/nmeth.4463.

miRcode	A map of putative microRNA target sites in the long non-coding transcriptome.	Jeggari, A., Marks, D.S., and Larsson, E. (2012). miRcode: a map of putative microRNA target sites in the long non-coding transcriptome. Bioinformatics 28(15), 2062-2063. doi: 10.1093/bioinformatics/bts344.
GSVA	gene set variation analysis for microarray and RNA-seq data.	Hänzelmann, S., Castelo, R., and Guinney, J. (2013). GSVA: gene set variation analysis for microarray and RNA-seq data. BMC Bioinformatics 14, 7. doi: 10.1186/1471-2105-14-7.
